# 2502 The need for an evidence-based CTS specific IDP for early career training and for a long-term and sustainable career in clinical translational sciences

**DOI:** 10.1017/cts.2018.230

**Published:** 2018-11-21

**Authors:** Camille A. Martina, Janice L. Gabrilove, Naomi Luban, Cecilia M. P. Sutton

**Affiliations:** University of Rochester Medical Center

## Abstract

OBJECTIVES/SPECIFIC AIMS: To establish a conceptual framework to develop a CTS-IDP with data analytics, and an e-Learning Faculty Development Guide on best practices and use of the IDP over the CTS academic life-course. METHODS/STUDY POPULATION: To accomplish our goal, we propose the following methods: (1) an online survey, using a convenience sample of the 24 KL2 CTSA IDP Collaborative members (conducted in 2017), to assess perceived needs for a universal CTS-IDP, current IDP practices, barriers to IDP use, and to discern and align each CTSA Hub’s interests, expertise and commitment to specific areas of the study; (2) A scoping narrative literature review, utilizing the Arksey and O’Malley framework covering the time period corresponding to the initiation of funding (1999) of the original K30 Clinical Research Curriculum Awards through to the present CTSA funding period, incorporating Medical Subject Heading (MeSH) keywords (career development; career development plan; employee plan; mentoring plans; compacts; research contracts; career planning; mentor guide), initially delineated by USC reference librarian and to be expanded by reference librarian services from the Icahn School of Medicine at Mount Sinai and University of Rochester, and performed on NIH searchable databases including NCBI PubMed, Central and Medline & Worldwide Science; Web of Science, ProQuest, ProQuest Abi/Inform, Google Scholar, Cochrane, Ovid MEDLINE databases, as well as Google for published papers in English and Spanish. For this portion of the work, we will describe and characterize (1) research career development or progression constructs, domains, and milestones; (2) establish the presence or absence of defined and/or pre-specified timed milestone objectives and inclusion of SWOT analytics (strengths, weaknesses, opportunities, and threats) and/or Gantt chart approaches; (3) delineate IDPs structure, toolkits and their key features (competencies, skills acquisition and processes utilized); (4) and identify specific gaps to best address the need for personalized career development education. Based on this review, we will synthesize CTS milestones, develop a time frame for meeting RCD expectations, and establish RCD benchmarks for achieving these milestones, all in consensus with the IDP Collaborative Workgroup. RESULTS/ANTICIPATED RESULTS: Seventy-seven percent of the IDP CTSA’s responded to the online survey, led by University of Rochester, and the results can be summarized as follows: (1) 100% agreed that the IDP process is important and should be considerably improved to optimize effectiveness; (2) a range of diverse IDP formats are utilized, making comparisons across programs difficult; (3) 50% of CTSA hubs report only fair to good compliance with the IDP process; (4) a major barrier to the IDP process is lack of instruction regarding how best to utilize; (5) poor alignment of currently available IDPs designed for basic science PhDs with CTS investigators; (6) an absence of a CTS specific IDP to best foster RCD for this specific career trajectory. When asked: What are the barriers to writing a detailed and thoughtful IDP, responses in order of agreement from greatest to least were: No verification of acquired competencies, beyond self-report (56%), Static platform (38%), Not constructed for clinical and translational researcher (31%), No analytical or documentation on use (31%), No instruction given to scholars on how to use it effectively and efficiently (31%), The IDP we are using is more constructed for PhD students and postdoctoral fellows (25%), No instruction given to the scholars on why it is important as adult learners (19%), and Not constructed for early career physicians/scientist (13%). Additional progress has been made on our Scoping review: An initial ABI/Inform and PubMed USC research librarian conducted search using Author names yielded 72 articles, of which only 2 were relevant to the topic at hand. A ProQuest™ search yielded 19 potentially relevant articles, 11 of which were of relevance to the topic of IDPs; and a Google Scholar search yielded 18 and 25 on career development and self-management, respectively. This has enabled us to put forth an initial model of factors that impact the purpose and design of IDPs that includes? DISCUSSION/SIGNIFICANCE OF IMPACT: Discussion: Our initial data suggests that many CTSA institutions see the need to further enhance the mentoring process with a more informed and personalized IDP template and process. Furthermore, our initial scoping review suggests a framework upon which to build specific components of a more ideal and useful IDP to best guide mentored research career development of CTS trainees. Significance: Developing and evaluating collaborative evidence-based CTS IDP and corresponding e-Learning Guide could potentially prevent or reduce important delays in RCD, a common roadblock for the translation of clinical interventions. Ultimately, the CTS-IDP serves not only to support and frame a scholar’s RCD “habits of mind” during training and early career development but to also to achieve a sustainable long-term career at a CTS researcher equipped to meet the ever challenging and dynamic research landscape.

